# Starvation Metabolism Adaptations in Tick Embryonic Cells BME26

**DOI:** 10.3390/ijms26010087

**Published:** 2024-12-26

**Authors:** Cintia Lopes Nogueira, Angélica F. Arcanjo, Maria Elisa Lima, Bruno Moraes, Renato Martins da Silva, Katia C. Gondim, Satoru Konnai, Isabela Ramos, Samara Santos, Alessandra D’Almeida Filardy, Kamila Guimarães Pinto, Itabajara da Silva Vaz Junior, Carlos Logullo

**Affiliations:** 1 Laboratório de Bioquímica de Artrópodes Hematófagos, Instituto de Bioquímica Médica Leopoldo de Meis, Universidade Federal do Rio de Janeiro, Rio de Janeiro 21941-590, Brazil; cintia.nogueira@bioqmed.ufrj.br (C.L.N.); angelica.arcanjo@bioqmed.ufrj.br (A.F.A.); maria.lima@bioqmed.ufrj.br (M.E.L.); bmarques@bioqmed.ufrj.br (B.M.); rjrenato@icloud.com (R.M.d.S.); isabela@bioqmed.ufrj.br (I.R.); 2 Laboratório de Bioquímica e Fisiologia de Insetos, Instituto de Bioquímica Médica Leopoldo de Meis, Universidade Federal do Rio de Janeiro, Rio de Janeiro 21941-617, Brazil; katia@bioqmed.ufrj.br (K.C.G.); samara.araujo@bioqmed.ufrj.br (S.S.); 3 Laboratory of Infectious Diseases, Hokkaido University, Sapporo 060-0810, Japan; konnai@vetmed.hokudai.ac.jp; 4 Laboratório de Imunologia Celular, Departamento de Imunologia, Instituto de Microbiologia Paulo de Goes, Universidade Federal do Rio de Janeiro, Rio de Janeiro 21941-617, Brazil; filardy@micro.ufrj.br (A.D.F.); guimaraespkamila@micro.ufrj.br (K.G.P.); 5 Centro de Biotecnologia and Faculdade de Veterinária, Universidade Federal do Rio Grande do Sul (UFRGS), Porto Alegre 91509-900, Brazil; itabajara.vaz@ufrgs.br

**Keywords:** starvation, *Rhipicephalus microplus*, energy metabolism

## Abstract

Ticks are hematophagous ectoparasites that transmit pathogens and inflict significant economic losses on the cattle industry. Remarkably, they can survive extended periods of starvation in the absence of a host. The primary objective of this study was to investigate the metabolic adaptations that enable the tick *Rhipicephalus microplus* to endure starvation using the BME26 cell line as a model system. To simulate nutrient deprivation, cells were subjected to starvation conditions by replacing the L-15 culture medium with phosphate-buffered saline (PBS). Our findings show that these tick cells can endure experimental starvation for up to 48 h. The assessment of glycogen levels in starved cells shows a significant decrease, at both the 24 h and 48 h marks. Additionally, upregulation of phosphoenolpyruvate carboxykinase (PEPCK) gene expression, along with downregulation of hexokinase (HK) and pyruvate kinase (PK) gene expression, indicated that BME26 cells would prioritize the gluconeogenic pathway over the glycolytic pathway under starvation conditions. Moreover, the transcriptional levels of autophagy-related genes (ATG) were upregulated in response to starvation. Taken together, our findings suggest a potential role for autophagy in supplying substrates for the gluconeogenic pathway in nutrient-deprived tick cells. This work contributes to the understanding of metabolic regulation in *R. microplus* ticks and offers valuable insights for tick control strategies.

## 1. Introduction

*Rhipicephalus microplus* is the most impactful tick affecting cattle, leading to substantial economic losses in the livestock industry [1]. Severe cattle tick infestations may result in weight loss and a subsequent decline in milk and meat production, as well as leather depreciation. Moreover, the *R. microplus* tick can also transmit pathogens such as *Babesia bovis*, *Babesia bigemina,* and *Anaplasma marginale*, etiological agents of bovine parasitic sadness [2,3]. The life cycle of *R. microplus* consists of two distinct phases: a free-living phase and a parasitic phase [4,5]. In the free-living phase, engorged females lay eggs that hatch in the environment and develop into larvae. During the parasitic phase, tick larvae attach to a host and feed on its blood while progressing through the nymphal and adult stages [5,6,7].

In the free-living phase, *R. microplus* embryo survival and development into larvae rely entirely on maternal nutrients that are deposited into the oocytes during the preoviposition phase [8,9,10]. Interestingly, *R. microplus* embryogenesis is known as an energy-intensive process that depends mostly on glycolytic and gluconeogenesis pathways [9,11]. However, tick eggs and larvae can survive without a blood meal for several months [12], which raises several questions regarding tick metabolism during starvation.

It is well known that the activation of gluconeogenesis pathways is one of the most common physiological strategies to cope with starvation [13]. In ticks, increased gluconeogenesis during the final stages of embryogenesis has been evidenced by the high activity of phosphoenolpyruvate carboxykinase (PEPCK) [11]. PEPCK plays a central role in gluconeogenesis and is widely regarded as the limiting step of this metabolic pathway [14]. It connects various metabolic pathways, with the synthesized phosphoenolpyruvate (PEP) involved in glyceroneogenesis and in the cataplerosis of the citric acid cycle [14,15,16]. Although gluconeogenic pathways are well characterized in different organisms, the mechanisms underlying tick gluconeogenesis activation and substrate sources during starvation are not fully understood. In this context, the BME26 cell line, derived from *R. microplus* embryos [17], emerged as a valuable model for investigating tick energetic metabolism and survival during starvation.

In the present work, BME26 cells were submitted to nutritional deprivation to elucidate the metabolic mechanisms underlying cell survival during starvation. We observed that starvation induces metabolic reprogramming in BME26 cells, including the activation of autophagic pathways as a potential source of product substrates for gluconeogenesis. These results contribute to our understanding of how *R. microplus* adjusts its metabolic processes to ensure metabolic integrity and development, even in the face of severe nutritional challenges. Moreover, obtaining insights into tick energy metabolism during starvation may be relevant for developing effective tick control strategies.

## 2. Results

### 2.1. BME26 Cells Tolerance to Starvation

**Figure 1:**
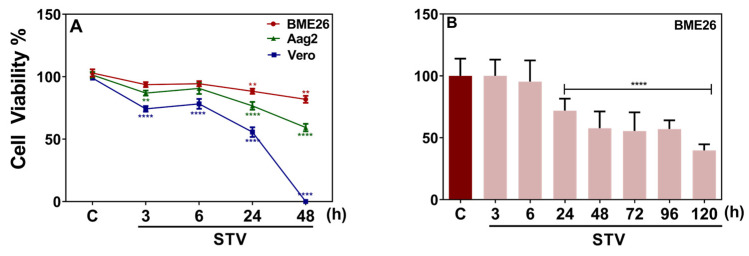
BME26 cells exhibit remarkable tolerance to nutrient deprivation over extended periods. (**A**) BME26, Aag2, and Vero cell viability was measured over time in response to starvation treatment (STV), using a Neubauer hemocytometer with the trypan blue exclusion technique. (**B**) MTT viability assay of BME26 cells over time in response to prolonged starvation treatment (STV). Control cells were maintained in L15 medium throughout the experiment. Three independent biological samples were tested in three experimental replicates each. Data are shown as mean ± SD, and were analyzed by (**A**) two-way ANOVA, followed by Tukey’s multiple comparisons test (**** *p* < 0.0001); (**B**) one-way ANOVA, followed by Bonferroni’s multiple comparisons test (** *p* < 0.01), (**** *p* < 0.0001).

To evaluate the response of BME26 embryonic tick cells to starvation conditions, we first conducted a comparative analysis including two other well-established cell lines: Aag2, derived from eggs of the mosquito *Aedes aegypti,* and Vero, from African green monkey kidneys. In these assays, Aag2 and BME26 viability remained consistently above 60% and 70%, respectively, even after 48 h of starvation, while Vero cells did not survive past 24 h (Figure 1A). These results indicate that the arthropod cells Aag2 and BME26 cells have a higher tolerance to starvation than the mammalian Vero cells. BME26 cells were also subjected to prolonged starvation, resulting in a 40% decrease in cell viability at 48 h (Figure 1B). Additionally, BME26 cell viability was measured after 24 h and 48 h of partial starvation, resulting in no significant impact on viability (Supplementary Figure S1).

**Figure 2:**
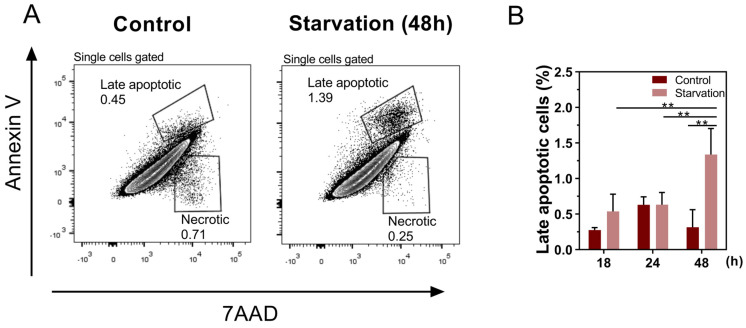
Starvation increases late apoptosis in embryonic tick cells BME26. Flow cytometry apoptosis assay: representative FACS plot and frequencies of (**A**) late apoptotic cells (Annexin V+ and 7AAD+) gated on single cells from control and starvation conditions. (**B**) Quantitative analysis of late apoptotic cells (Annexin V+ and 7AAD+), gated on single cells, under control and starvation conditions. Data are representative of three independent experiments with similar results. Data are shown as mean ± SD and were analyzed by one-way ANOVA with Tukey’s multiple comparisons test; ** *p* < 0.01.

To better understand the impact of starvation on BME26 cell viability, we used flow cytometry to analyze cell death dynamics under nutrient-restrictive conditions. No significant differences were found in overall levels of apoptotic cell death between the starved and control groups. However, starvation induced significantly higher levels of late apoptotic cells compared with control after 48 h (*p* < 0.01) (Figure 2A,B).

**Figure 3:**
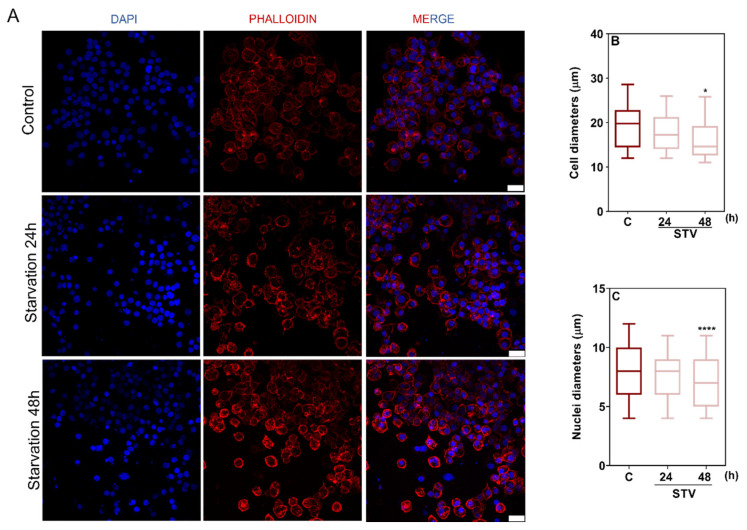
Prolonged starvation causes morphological changes in BME26 cells. (**A**) Actin cytoskeletal morphology (red) and nuclei (blue) were observed after starvation treatment (STV). Control cells were maintained in L15 medium. Scale bar: 20 µm. (**B**) Quantitative analysis of cell diameter after starvation for 24 h and 48 h. (**C**) Quantitative analysis of the maximum diameter of nuclei after starvation (STV); results were obtained from 300 to 400 nuclei. The graphs show the medians and min to max values of at least 60 cells per condition, compared by the Kruskal-Wallis test followed by Dunn’s post hoc test (* *p* < 0.01), (**** *p* > 0.0001).

Morphological changes in BME26 cells after 24 h or 48 h of starvation were evaluated using confocal fluorescence microscopy (Figure 3A). At 24 h, cells did not present differences in morphology (Figure 3). On the other hand, cells starved for 48 h were smaller (*p* > 0.01 for cell diameter) (Figure 3B) and had smaller nuclei (*p* > 0.0001 for nucleus diameter) (Figure 3C).

Overall, it was demonstrated that BME26 cells are resistant to starvation for at least up to 48 h and present subtle morphological changes that indicate cell death by late apoptosis. These findings highlight the potential link between resistance to starvation and the regulation of apoptotic processes in these cells.

### 2.2. Effects of Starvation on Nutritional Reserves

**Figure 4:**
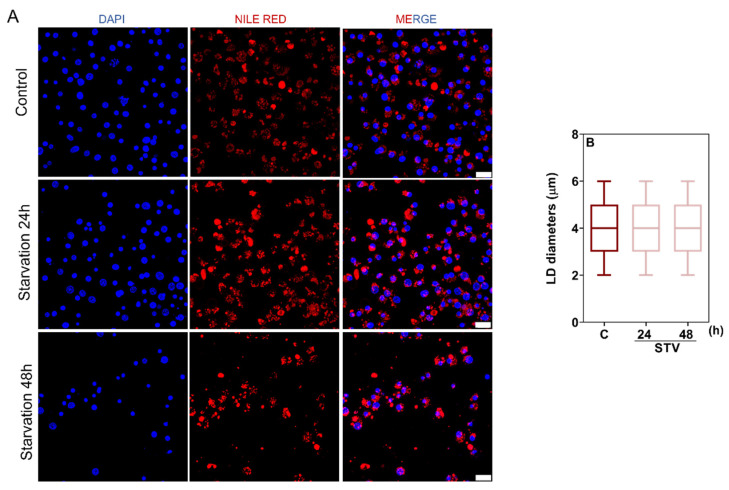
Quantitative assessment of lipid droplet dynamics shows no impact of starvation treatment. (**A**) Cells were stained with DAPI to label nuclei (blue) and Nile Red to stain lipid droplets (red) and subsequently observed under a laser scanning confocal microscope. Scale bar: 20 µm. (**B**) Maximum diameter of lipid droplets (LD) was determined from three independent experiments. Results were obtained from 208 to 301 LDs per group. The graph shows the medians and Min to Max values of at least 60 cells per condition. Statistical analysis was performed using the Kruskal–Wallis test followed by Dunn’s post hoc test.

To investigate the mobilization of energy reserves in BME26 cells under starvation, we performed confocal microscopy to visualize lipid droplets (LD). As shown in Figure 4A, non-starved cells exhibited well-distributed lipid droplets. After 24 h and 48 h of starvation, there was no significant change in lipid droplet diameter (Figure 4B). The relative frequency of lipid droplets also did not present significant differences in distribution (Supplementary Figure S2).

**Figure 5:**
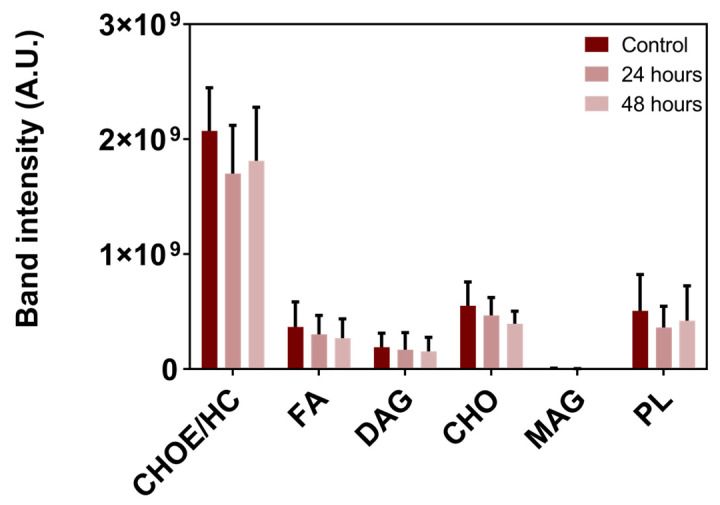
Lipid composition in BME26 cells is not affected by starvation. Lipid composition was assessed by TLC followed by densitometry. CHOE: cholesteryl ester; HC: hydrocarbon; FA: fatty acid; DAG: diacylglycerol; CHO: cholesterol; MAG: monoacylglycerol; PL: phospholipids. Control cells were maintained in L15 medium. Starvation treatment was performed for 24 h and 48 h. The graphs show the mean ± SD of three independent experiments (*n* = 3 replicates each), compared by one-way ANOVA followed by Tukey’s post hoc test.

Thin-layer chromatography (TLC) analysis of the lipid composition in BME26 cells revealed that the content of the major observed classes of lipids (cholesteryl ester/hydrocarbons, fatty acids, diacylglycerol, cholesterol, monoacylglycerol, and phospholipids) was not significantly affected after 24 h or 48 h of starvation (Figure 5). These results suggest that BME26 cells are resistant to short-term starvation with no impact on lipid reserves.

**Figure 6:**
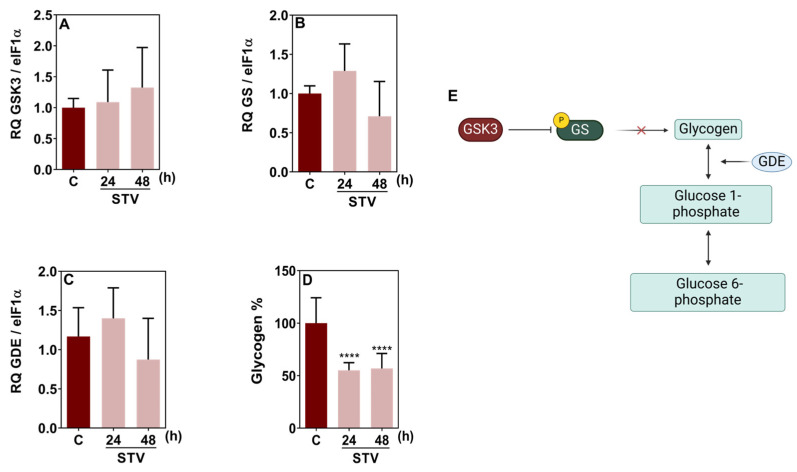
Starvation does not change the relative gene expression of key enzymes in glycogen metabolism but reduces glycogen reserves in BME26 embryonic tick cells. Gene transcription of (**A**) glycogen synthase kinase 3, (**B**) glycogen synthase, and (**C**) glycogen debranching enzyme. (**D**) Glycogen levels. (**E**) Illustration of the regulatory mechanisms involved in glycogen synthesis. The experiment was performed with three independent biological samples in three experimental replicates each. Data are shown as mean ± SD and were analyzed by one-way ANOVA, compared to control in Tukey’s multiple comparisons test (**** *p* < 0.0001).

To assess the mobilization of glycogen in BME26 cells under starvation, we analyzed the expression of key genes involved in glycogen metabolism and measured glycogen content (Figure 6). No significant changes between the starved and control groups were detected for glycogen synthase kinase 3 (GSK3), glycogen synthase (GS), and glycogen debranching enzyme (GDE) gene transcription (Figure 6A–C). However, a decrease in glycogen content was observed after 24 h and 48 h of starvation, as indicated by the significant reduction in glycogen percentage compared to the control group (Figure 6D). These results suggest that, despite the expression of key enzymes involved in glycogen metabolism remaining stable, mobilization of glycogen reserves triggered by starvation in BME26 cells was evident at both tested time points. A schematic representation of glycogen metabolism is presented in Figure 6E.

### 2.3. Partial Gluconeogenesis as a Metabolic Strategy for Starvation Resistance in Tick Cells

**Figure 7:**
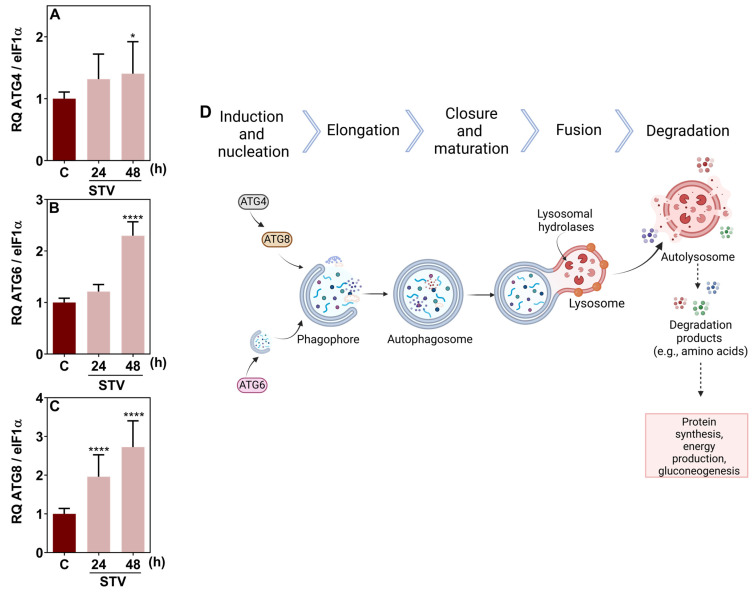
Autophagic response to starvation in BME26 cells. Transcription of (**A**) ATG8, (**B**) ATG4, and (**C**) ATG6, measured after 24 h and 48 h of starvation (STV). (**D**) Schematic diagram of the autophagy process. The experiment was performed with three independent biological samples in three experimental replicates each. Data are shown as mean ± SD, and were analyzed by one-way ANOVA, compared to control in Tukey’s multiple comparisons test (* *p* < 0.05), (**** *p* > 0.0001).

To understand the tick cellular response to starvation, we also examined the expression of relevant autophagy-related genes, along with genes encoding key glycolytic and gluconeogenic enzymes in BME26 cells. Figure 7 shows the relative quantification of transcripts of the autophagy genes ATG4, ATG6, and ATG8. The analysis revealed a significant upregulation in the transcription of all three genes after 24 h and 48 h of starvation, indicating enhanced autophagic activity. Figure 7D schematically illustrates the stages of autophagy, including induction, nucleation, elongation, closure, fusion, and degradation, highlighting the role of autophagy in recycling cellular components to provide nutrients.

**Figure 8:**
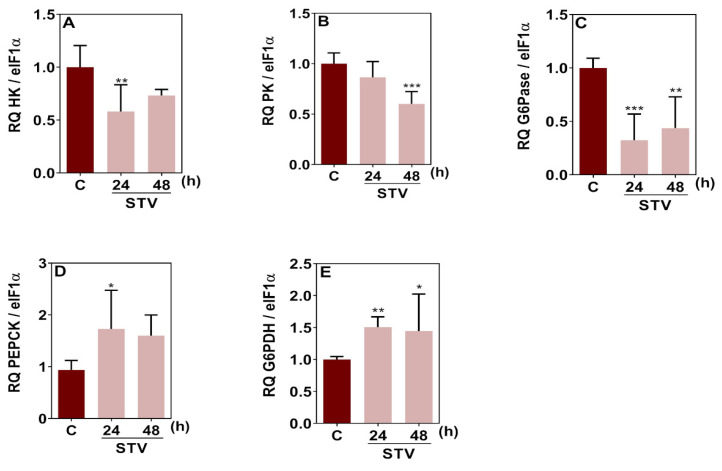
Relative transcription of key gluconeogenesis genes in BME26 cells is modulated by starvation treatment. (**A**) HK—Hexokinase, (**B**) PK—Pyruvate Kinase, (**C**) G6Pase—Glucose-6-Phosphatase, (**D**) PEPCK—Phosphoenolpyruvate Carboxykinase, (**E**) G6PDH—Glucose-6-Phosphate Dehydrogenase. Transcript levels were measured at 24 and 48 h of starvation (STV). The experiment was performed with three independent biological samples in three experimental replicates each. Data are shown as mean ± SD and were analyzed by one-way ANOVA, compared to control in Tukey’s multiple comparisons test (* *p* < 0.05, ** *p* < 0.01, *** *p* < 0.001).

The relative transcript levels of the glycolytic and gluconeogenic genes are presented in Figure 8. The results indicated a significant downregulation of hexokinase (HK) and pyruvate kinase (PK) genes after 24 h and 48 h of starvation, respectively, suggesting a decrease in glycolytic flux (Figure 8A, B). Additionally, glucose-6-phosphatase (G6Pase) gene transcription was significantly reduced at both 24 h and 48 h timepoints (Figure 8C). On the other hand, the transcription of PEPCK gene was significantly upregulated at 24 h of starvation (Figure 8D), suggesting an increase in gluconeogenesis. Moreover, the relative transcript levels of glucose-6-phosphate dehydrogenase (G6PDH) gene also showed a significant increase (Figure 8E), suggesting a potential shift toward the pentose phosphate pathway (PPP) for maintaining redox balance and biosynthesis during starvation.

**Figure 9:**
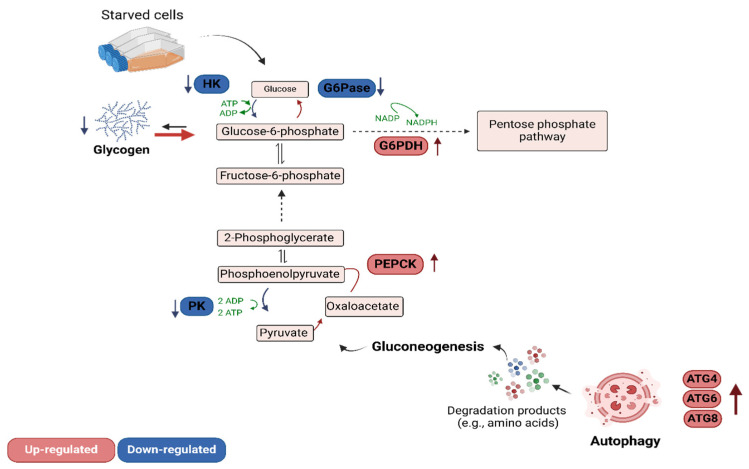
Metabolic pathways in starving BME26 cells: Key regulatory mechanisms in response to nutrient deprivation. Glycogen degradation is upregulated, and gluconeogenesis is activated, with PEPCK (upregulated) and PK (downregulated) playing crucial roles. Metabolite flow is directed toward the pentose phosphate pathway, and autophagy is induced, as indicated by the upregulation of ATG4, ATG6, and ATG8.

Overall, the coordinated increase in autophagic activity and in the transcription of gluconeogenic enzyme genes in starved BME26 cells indicates a metabolic adaptation to nutrient deprivation. The downregulation of glycolytic enzymes and upregulation of gluconeogenic pathways, along with a shift toward the pentose phosphate pathway, may reflect a strategic reprogramming of cellular metabolism aimed at maintaining energy homeostasis and redox balance (Figure 9).

## 3. Discussion

Mechanisms for maintaining metabolic homeostasis during periods of nutritional deprivation are essential for ensuring proper cellular function and sustaining cell viability [18,19]. Among these mechanisms, the use of alternative sources of energy, glycogenolysis, autophagy, and gluconeogenesis are all conserved pathways that can be used in cellular adaptation to starvation [12,19,20]. These metabolic pathways are of special interest in the context of the free-living phase of *R. microplus* life cycle, considering its ability to survive prolonged periods of starvation.

To better understand this tick’s remarkable adaptability to starvation, this study investigated the metabolic remodeling processes undergone by *R. microplus* cells under experimental starvation conditions. The BME26 cell line, derived from *R. microplus* embryos, has been used as a model system for studying tick metabolism, development, and control strategies under laboratory conditions [21,22,23,24]. Due to their embryonic origin, BME26 cells may also be used as a model of the free-living stages of tick development. Here, we demonstrated that these cells are able to cope with intense nutritional deprivation induced by incubation in PBS, maintaining cell viability for more than 24 h. Indeed, BME26 cells exhibited resistance to short-term starvation, maintaining cytoskeletal integrity and cell size within the first 24 h, as evidenced by the stable organization of actin filaments and consistent nuclear morphology. However, a small population of cells displaying late apoptotic features was detected after 48 h of starvation, suggesting that prolonged deprivation eventually overcomes BME26 adaptive mechanisms, leading to increased apoptosis. Interestingly, starvation-induced apoptosis was also observed in other arthropod cell lines over 48 h [25], which agrees with the observed BME26 cells’ short-term starvation tolerance.

Arthropods use various strategies to cope with starvation; most insights derive from studies on *Drosophila melanogaster*, which are not particularly known for exceptional starvation tolerance [26]. Prolonged starvation or selection for resistance in these fruit flies leads to systematic changes in energy reserves [27,28]. However, specific changes in energy reserves during starvation are unknown in ticks. To investigate the metabolic reserves potentially utilized by ticks during prolonged starvation, we conducted an analysis of lipid metabolism in BME26 cells under starvation conditions. Lipid droplet dynamics remain unaffected during the initial 24 h period and up to 48 h of starvation, with stable levels of different lipid classes. These findings indicate that BME26 cells maintain lipid homeostasis and do not utilize lipid metabolism as an immediate or delayed response to starvation.

In most animals, metabolism tends to be reprogrammed during the course of a starvation period, shifting from the breakdown of carbohydrates to lipolysis and finally to proteolysis [18,19]. Our results demonstrated that a similar response occurs in the BME26 cell model. We demonstrated that, despite the stable expression of GSK3, GDE, and GS genes, there is a significant decrease in glycogen content after 24 h and 48 h of starvation, suggesting that BME26 cells prioritize glycogen utilization over synthesis during nutrient scarcity. This finding is supported by the fact that GSK3 regulates GS activity post-translationally through phosphorylation, which might not necessarily require changes in gene expression levels [29]. The observation that GDE gene expression remains unaltered by starvation further indicates that the regulation of glycogen breakdown may rely more on the activity of existing enzymes rather than new synthesis. Moreover, the reduction of glycogen levels highlights the role of glycogen as an energy source during starvation, enabling BME26 cells to meet energy demands under stress conditions. These findings emphasize BME26 metabolic flexibility and the ability to adapt to nutrient deprivation by effectively utilizing glycogen stores, ensuring cell survival. Further investigation of glycogenic enzyme activity levels is necessary to provide a more comprehensive understanding of the regulatory mechanisms underlying tick glycogen metabolism in response to starvation.

In addition to glycogen consumption, autophagy is another important cellular process that supports homeostasis by degrading and recycling cytoplasmic components within lysosomes, thus acting as a potential supplier of gluconeogenesis substrates [30,31,32]. Autophagy is driven by a group of evolutionarily conserved genes known as autophagy-related genes (ATGs). Many ATG genes and their associated proteins have been identified, each playing specific roles in regulating the various stages and forms of autophagy [30,32,33]. ATG8 is thought to facilitate the expansion and closure of the autophagosomal membrane [34]. Previous studies have shown that embryonic cells from *Ixodes scapularis* and *R. microplus* can undergo autophagy as a response to short-term starvation [20,33]. It was also revealed that the ATG gene superfamily is well conserved in ticks and plays a role in autophagy pathways triggered by amino acid deprivation [20]. Consistent with these findings, our study observed a significant upregulation of key autophagy-related genes, including ATG4, ATG6, and ATG8, in BME26 cells subjected to prolonged starvation (48 h). These results suggest that BME26 cells may maintain their viability through the activation of autophagy.

Along with autophagy activation, we also demonstrated the activation of gluconeogenesis, mostly evidenced by the increased PEPCK gene expression in BME26 cells under prolonged starvation. Moreover, a potential shift from glycolysis to the pentose phosphate pathway during starvation was indicated by increased levels of G6PDH gene expression. We propose that the pentose phosphate pathway and gluconeogenesis contribute to these mechanisms of cellular adaptation to starvation, and they do so by ensuring a continuous supply of glucose to meet energy demands of BME26 cells under prolonged nutrient-limited conditions.

In conclusion, our study sheds light on the dynamic regulation of autophagy and gluconeogenesis in BME26 cells during starvation, which is summarized in Figure 9. Our findings suggest that the BME26 cell line is a viable model for studying tick metabolism under starvation. However, further studies are needed to fully elucidate tick metabolic reprogramming in vivo. Overall, the data indicate that increasing autophagic activity supports gluconeogenesis by recycling cellular components, thereby maintaining energy homeostasis and ensuring cell survival when nutrients are depleted. Our study provides valuable insights into the metabolic flexibility and resilience of tick embryonic cells and their adaptations to environmental challenges. Importantly, these results also add to the growing knowledge base that aids in the discovery of new and more effective strategies for tick prevention and control.

## 4. Materials and Methods

### 4.1. Cell Lines and Experimental Starvation

The BME26 tick embryo cell line was originally obtained as previously described [17] and maintained according to the established protocols [23]. Additionally, other invertebrate and vertebrate cell lines (Aag2 and Vero, respectively) were used in comparative analysis. Aag2 cells were maintained as previously described [23]. Vero cells were cultured in Dulbecco’s Modified Eagle’s Medium (DMEM) supplemented with 10% fetal bovine serum (heat-inactivated) and 1% penicillin-streptomycin, all obtained from Gibco BRL (Grand Island, NY, USA), at 37 °C in a humidified, 5% CO_2_ atmosphere.

For starvation experiments, BME26 and Aag2 cells were counted, resuspended, and seeded at a density of 5 × 10^5^ cells per well in 500 µL of medium in a 24-well plate. The cells were incubated overnight at 28 °C to allow adhesion. The medium was then replaced by phosphate-buffered saline (PBS, pH 7.4), and the cells were kept under these starvation conditions for 24 or 48 h prior to further analysis. For partial starvation, the same seeding procedure was performed, but after adhesion the medium was replaced by PBS containing 10% heat-inactivated fetal bovine serum (Gibco BRL, Grand Island, NY, USA). A similar protocol was applied to Vero cells, with minor modifications: Cells were incubated at 37 °C in a humidified atmosphere with 5% CO_2_ to allow adhesion before starvation treatment.

### 4.2. Assessment of Cell Viability

Cell viability was assessed using a Neubauer hemocytometer and the 0.04% Trypan Blue (Sigma-Aldrich, Saint Louis, MO, USA) exclusion assay, with viability determined by visual inspection [35]. The experimental procedure and calculations were performed following standard methodology, as previously reported [23]. Cell viability was alternatively assessed using the 3-(4,5-dimethylthiazol-2-yl)-2,5-diphenyltetrazolium bromide (MTT) (Sigma-Aldrich, Saint Louis, USA) colorimetric assay, performed on 24-well plates as described previously [23,36]. Absorbance values at 570 nm from the control condition were used for normalization (considered as 100% viability).

### 4.3. Flow Cytometry

After starvation treatment, cells were suspended and incubated with Annexin V antibody (BD Pharmingen, cat# 556419, Chicago, IL, USA) for 15 min at room temperature, and then with 7AAD (BD Pharmingen, cat# 559925) for 10 min at room temperature, for apoptosis assessment. Data were acquired on a BD LSR Fortessa flow cytometer (BD Biosciences, Chicago, IL, USA) using BD FACS Diva software version 8.0.3 (BD Biosciences, Chicago, IL, USA). Compensation and data analysis were performed using FlowJo software vX.0.7 (TreeStar, Ashland, OR, USA). Late apoptotic cells were identified as AnnexinV + 7AAD+.

### 4.4. Total RNA Extraction, cDNA Synthesis, Real-Time Quantitative PCR, and Relative Quantification Analyses

Total RNA was extracted from starved or control BME26 cells harvested from 24-well plates using the Trizol reagent (Invitrogen, Grand Island, NY, USA) according to the manufacturer’s instructions. The cDNA synthesis reaction was performed in the presence of RT random primer and reverse transcriptase using the High-Capacity cDNA Reverse Transcription kit (Applied Biosystems), according to the manufacturer’s instructions. After the reverse transcription reaction, the product of each sample was used for the qPCR reaction. The qPCR was performed using a StepOne Plus Real-Time PCR equipment (Applied Biosystems, Inc., Foster City, CA, USA) with SYBR Green PCR master mix (Applied Biosystems, Inc., Foster City, CA, USA) using the primers from each specific gene (listed in Supplementary Table S1). Relative expression was determined using the Ct values from each analysis in the Relative Expression Software Tool’s table (Step One Software 2.3 Version) [37]. The elongation factor 1α (elf1α) was used as a housekeeping reference gene [38].

### 4.5. Glycogen Quantification

Glycogen quantification in BME26 cells under starvation was performed as previously described [21], with minor modifications. Briefly, 3 × 10^6^ cells were used for each condition tested, and glycogen levels were normalized based on the total cell count for each condition.

### 4.6. Lipid Separation by Thin Layer Chromatography (TLC)

Cells were subjected to lipid extraction [39] followed by lipid separation by thin-layer chromatography (TLC) on a silica gel plate (Merck KGaA, Darmstadt, Germany) using two consecutive solvent systems [40]. The plates were stained with 10% cupric sulfate (*w/v*) in 8% phosphoric acid (*v/v*); lipid classes were identified by comparison with commercial standards (Sigma-Aldrich, Saint Louis, MO, USA), and lipid composition was determined by densitometry using ImageJ software version 1.50i (NIH Image, Bethesda, MD, USA).

### 4.7. Microscopy

A sample of 2 × 10^5^ cells was seeded in 500 µL of culture medium in a cell culture dish and incubated at 28 °C in complete medium for 24 h to promote cell adhesion. Subsequently, the cells underwent starvation treatment as described in Section 4.1, after which they were stained to analyze lipid droplets using Nile Red. Staining consisted of incubation for 10 min in a 0.001% Nile Red solution (Sigma-Aldrich, Saint Louis, MO, USA) prepared in 75% glycerol. The stained cells were then mounted in 100% glycerol on glass slides and immediately imaged with excitation/emission wavelengths of 543 nm. The average diameters of the lipid droplets were quantified from four to five representative images per group, across three independent experiments. The measurements were performed using DAIME image analysis software (2.2 version) [41] with automatic edge detection and segmentation to ensure precise droplet identification. In addition, phalloidin (Invitrogen, Grand Island, NY, USA) and DAPI (Thermo Fisher Scientific, Waltham, MA, USA) were used for cellular morphology evaluation, as previously described [42]. The cells were analyzed in a laser scanning confocal microscope, model Zeiss LSM710 (Carl Zeiss, Oberkochen, Germany).

### 4.8. Statistical Analyses

The experiments were carried out with three independent biological samples, with three experimental replicates each. The results were analyzed by one-way ANOVA followed by Tukey’s test to compare more than two conditions. For the viability assay, two-way ANOVA and Tukey’s multiple comparisons test were used. A Kruskal–Wallis test followed by Dunn’s test was used to compare the diameter of cells, LDs, and nuclei. All statistical analyses were performed using Prism 6.0 (GraphPad Software Inc., La Jolla, CA, USA).

### 4.9. Ethical Approval and Compliance with Institutional Guidelines

All experimental protocols were conducted following guidelines from the ethical committee on animal use for research at the Universidade Federal do Rio de Janeiro (CEUA-UFRJ), as approved under process number 01200.001568/2013-87.

## Data Availability

All data are included in the article and Supplementary Materials.

## Supplementary Materials

The following supporting information can be downloaded at: https://www.mdpi.com/article/10.3390/ijms26010087/s1.
