# Supplementary material for: Starvation Metabolism Adaptations in Tick Embryonic Cells BME26
Source: Int J Mol Sci. 2024 Dec 26;26(1):87. doi: 10.3390/ijms26010087 (PMC11719990; doi:10.3390/ijms26010087)
Supplement: Supplementary file 1 [file ijms-26-00087-s001.zip › Supplementary table.pdf]

Supplementary Table S1. Primers used in qPCR analysis of *Rhipicephalus microplus* with the respective GenBank accession numbers:

Gene	Primers Used	GenBank Access Number
hexokinase (HK)	5'- CATGGACAAAGAGCTTCAACTGCTC-3'<br>5'- GGAAAGCTCCCTTGACCAGGGTA-3'	KF951259
pyruvate kinase (PK)	5'-GGGCAAGAGGGCAAGACAACTG-3'<br>5'- CACGTTGAGCACCTTGGTGATG-3'	KF951260
glucose-6- phosphatase (G6Pase)	5'-GGCAGCCATTTGGTACATCATCC-3'<br>5'- CGACAGGCTGACAATGCACAGG-3'	KF951262
phophoenolpyruvate carboxykinase (PEPCK)	5'-AGGAGAAGCAGCGCCTGT-3'<br>5'-GTGGTTGTTCTCGGCCTCG-3'	EF142067.1
glycogen synthase (GS)	5'-GCTGGTATCGGGCTGATCCTG-3'<br>5'-GATGCCTCTGTCTCCAGCCTCC-3'	KF951264
glycogen synthase kinase 3 (GSK3)	5'-CCCACACCCGCTATTTATTG-3'<br>5'- TGTGCAGGAGAGCCAGTTTA-3'	EF142066
glycogen-debranching enzyme (GDE)	5'-ATGCTCAGGATCACGCAGAAGC-3'<br>5'-GTACGTCGGTTGGGAAGGACAAGG-3'	XM_037432504
glucose-6- phosphate Dehydrogenase (G6PDH)	5'-CGCAACGAATTGGTATTGAGG-3'<br>5'-CGACTGCCATAGGTGAGATCC-3'	EU595878.1
isocitrate dehydrogenase (IDH)	5'-CTTCAAAGCAGGTCTTATGG-3'<br>5'-AGGAACGGGAATATCAACTC -3'	KY953209
elongation factor 1-alpha (ELF1α)	5′-CGTCTACAAGATTGGTGGCATT-3′<br>5′-CTCAGTGGTCAGGTTGGCAG-3′	EW679365
BmeATG6	5′ GAATTTGGTGTCCCACAGGAA 3′<br>5′ TGCGGAACTGCCCCTGTAC 3′	KY660684
BmeATG8	5'-GAGCGAGGGTGACAAGATTC- 3'<br>5'-TGGCACTAGTAGGGGGAATG-3'	KY660686
BmeATG4	5′-AATCGGATGCAGCCACATG-3′<br>5′-GAGCACAAGAGGCTTCCAAGA-3′	KY660682
