# Supplementary figures and images for: Starvation Metabolism Adaptations in Tick Embryonic Cells BME26
Source: Int J Mol Sci. 2024 Dec 26;26(1):87. doi: 10.3390/ijms26010087 (PMC11719990; doi:10.3390/ijms26010087)

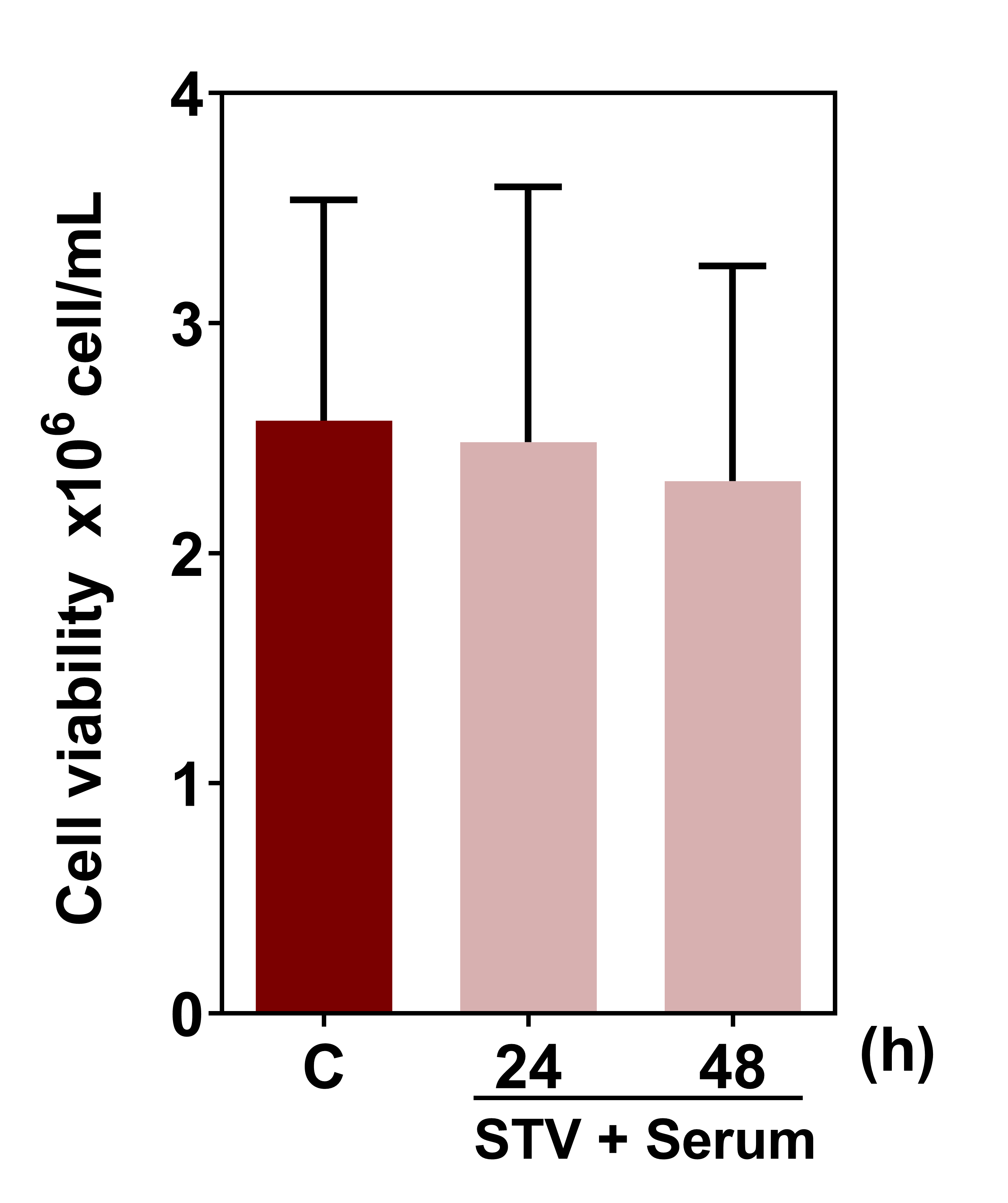

Supplement: Supplementary file 1 [file ijms-26-00087-s001.zip › Supplementary Figure S1.tif]

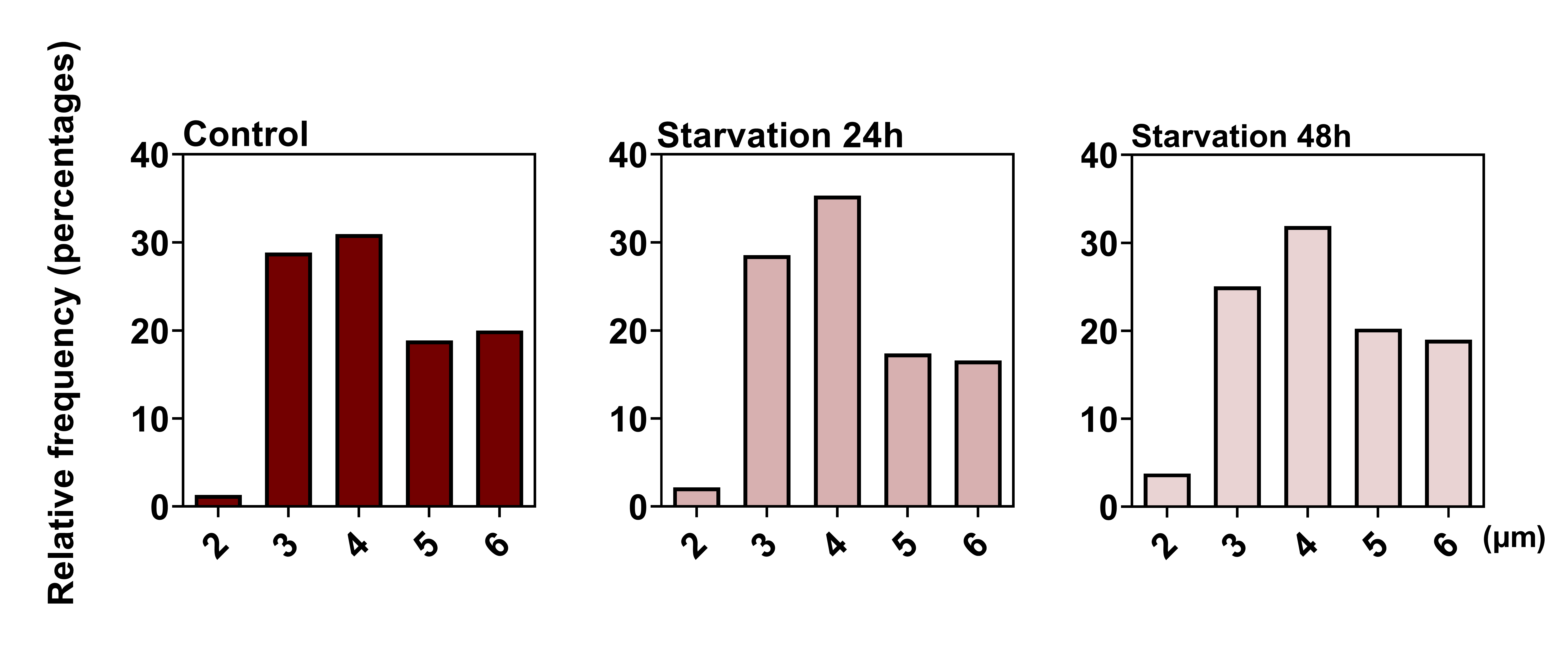

Supplement: Supplementary file 1 [file ijms-26-00087-s001.zip › Supplementary Figure S2.tif]
